# Lipoxin A4 Restores Septic Renal Function *via* Blocking Crosstalk Between Inflammation and Premature Senescence

**DOI:** 10.3389/fimmu.2021.637753

**Published:** 2021-04-15

**Authors:** Chaojin Chen, Rongzong Qiu, Jing Yang, Qian Zhang, Guoliang Sun, Xiaofeng Gao, Ziqing Hei, Haocong Ji

**Affiliations:** ^1^ Department of Anesthesiology, The Third Affiliated Hospital, Sun Yat-Sen University, Guangzhou, China; ^2^ Department of Anesthesiology, Guangdong Medical University, HuiZhou First Hospital, Huizhou, China

**Keywords:** acute kidney injury, sepsis, lipoxin A4, inflammation resolution, cellular senescence

## Abstract

Acute kidney injury (AKI) occurs in half of patients with septic shock, resulting in unacceptably high mortality. However, effective preventive treatments are still lacking. We hypothesized that pretreatment with lipoxin A4 (LXA4), known to promote inflammation resolution, may attenuate septic AKI *via* blocking crosstalk between inflammation and cellular senescence. In this study, rats developed AKI following cecal ligation and puncture (CLP), as evidenced by a dynamic increase in serum creatinine, blood urea nitrogen, urinary kidney injury molecule-1, neutrophil gelatinase-associated lipocalin, and pathological injury, accompanied by increased levels of inflammation (IL-6, TNF-α, and HMGB1) and tubular cell senescence. While, on the one hand, inhibition of senescence with rapamycin restored renal function and attenuated septic inflammatory response, on the other hand, LXA4 administration inhibited renal inflammation and tubular epithelial cell senescence after CLP. Ultimately, pretreatment with LXA4 significantly restored renal function and increased the survival rate of rats after CLP. Furthermore, LXA4 inhibited NF-κB-mediated inflammatory response and the p53/p21 senescence pathway *in vivo* and *in vitro*. However, the effect was reversed by PPAR-γ siRNA and antagonist. These results indicated that LXA4 exerted its renoprotective effects by blocking the crosstalk between inflammation and premature senescence in a PPAR-γ-dependent manner. Our findings also suggested that premature senescence plays a critical role in septic AKI and that inhibition of the crosstalk between inflammation and premature senescence may represent a new and major mechanism through which LXA4 attenuates septic AKI.

## Introduction

Sepsis is a life-threatening organ dysfunction caused by systemic infection (1), and accounted for 11 million deaths worldwide in 2017 (2). More than 50% of patients with sepsis develop acute kidney injury (AKI), which is considered a critical pathophysiology of sepsis (3). Therefore, targeting septic AKI is of great significance in reducing the mortality associated with sepsis.

Increasing evidence indicates that the pathogenesis of AKI with sepsis is complex and multifactorial, involving the interplay among oxidative stress, inflammation and cellular injury (4). Notably, premature cell senescence has emerged as an important feature of AKI (5). Acute DNA damage response has been found to induce inflammation and senescence, and is also called stress-induced premature senescence (SIPS) (6). Evidence suggested that renal ischemia/reperfusion (IR) induced cell division arrest at the G2/M phase and made a population of renal tubular epithelial cells become senescent (7). Our earlier study also showed that sepsis causes renal tubular cell senescence (8). However, the role of renal tubular cell senescence in septic AKI is largely unknown.

Increasing number of studies have shown a crosstalk between inflammation and senescence in various diseases (9). On the one hand, excessive inflammatory response has been widely reported to be one of the main causes of SIPS (10–13). On the other hand, senescent cells are known to induce senescence of their neighboring cells through the senescence-associated secretory phenotype (SASP) that involves inflammatory cytokines and chemokines to amplify the subsequent inflammatory response (9). As septic AKI occurs *via* a network of pro-inflammatory pathways that purportedly interact with cell senescence (8, 14, 15), whether blocking the crosstalk between inflammation and senescence can restore septic renal function has not been explored previously.

Although much effort has focused on developing therapeutic targets for septic AKI, effective treatments are still lacking. Lipoxin A4 (LXA4) is one of the most important mediators that resolves inflammation by activating anti-inflammatory pathways at the physiological end of acute inflammatory phase (16). A previous study showed that LXA4 reduced NF-κB-mediated pro-inflammatory gene expression in a receptor-dependent manner (17). Dunn et al. reported that activation of LXA4 signaling served as a potential therapeutic target for aging-related chronic inflammation and Alzheimer’s disease (18). As a ligand of peroxisome proliferator activated receptor (PPAR), LXA4 has also been reported to mediate PPAR-γ-dependent resolution of inflammation in experimental stroke (19) and *in vitro* renal tubular epithelial cell hypoxia/reoxygenation injury model (20). However, whether exogenous LXA4 supplementation participates in the process of resolution of inflammation in septic AKI *in vivo* and the underlying mechanisms of LXA4-related renoprotection need to be clarified.

This study aimed to investigate the underlying mechanism of the crosstalk between senescence and inflammation in AKI caused by sepsis. Considering the properties of LXA4 that have been showed in previous researches, we hypothesized that LXA4 treatment may block the interplay between inflammatory reaction and cell senescence in septic kidney tissues. Furthermore, we investigated whether LXA4 can attenuate cecal ligation and puncture (CLP)-induced AKI by inhibiting the NF-κB network in a PPAR-γ-dependent manner.

## Materials and Methods

### Reagents

LXA4 was purchased from GLPBIO (Montclair, California, US). Cellular senescence assay, rapamycin, Boc-2 (C44H59N5O8), GW9662, rosiglitazone (RSG), and cellular senescence assay kit were purchased from Sigma-Aldrich (Milwaukee, Wisconsin, US). Kidney injury molecule-1 (KIM-1), neutrophil gelatinase-associated lipocalin (NGAL), interleukin-6 (IL-6), tumor necrosis factor - α (TNF-α), high mobility group box 1(HGMB1), and LXA4 enzyme-linked immunosorbent assay (ELISA) kits were all purchased from USCN (Wuhan, China). PPAR-γ and p21 antibody, and PPAR-γ transcription factor assay kit were purchased from Abcam (Cambridge, UK). p-p65 and p53 antibodies were purchased from CST (Massachusetts, US).

### Animals

Male Sprague-Dawley rats (220–250 g) were obtained from Guangdong Medical Laboratory Animal Center (Guangzhou, China). All the rats were housed under standard conditions (25°C with 12 h light-dark cycles) and were fasted with distilled water and specific pathogen-free food. The experimental protocol used was approved by the Institutional Animal Care and Use Committee at the Third Affiliated Hospital of Sun Yat-sen University, and performed in accordance with the National Institutes of Health guidelines for the use of experimental animals.

### CLP Model

All rats in model groups were anesthetized by isoflurane inhalation. The anesthetized rats received midline laparotomy and the cecum was separated, ligated below the ileocecal valve, and punctured twice with a No. 20 steel needle. A heating pad was used to maintain the body temperature at 36–38°C during the surgery. Following that, all the rats were subjected to fluid subcutaneous resuscitation and returned to their cage.

### Treatment Protocol

To explore the dynamic changes of septic AKI, a total of 60 rats were distributed into five groups randomly (n = 12 per group): sham-operated, CLP 6-, 12-, 18-, and 24-h groups. Subsequent *in vivo* studies were performed using the 18 h CLP model.

To explore the underlying mechanisms involved in septic AKI, a total of 80 rats were randomly divided into eight groups (n = 10 per group): Sham group, CLP group, rapamycin pretreatment group (pre-injected intraperitoneally with 1 mg/kg rapamycin for 1 h with or without CLP), LXA4 pretreatment group (pre-injected intravenously with 100 µg/kg LXA4 for 30 min prior to CLP), LXA4 + Boc-2 pretreatment group (intravenously pre-injected with 100 µg/kg LXA4 for 30 min and 50 mg/kg Boc-2 for 20 min prior CLP), LXA4 + GW9662 pretreatment group (intravenously pre-injected 100 µg/kg LXA4 for 30 min, and 1 mg/kg GW9662 for 20 min prior CLP), LXA4 + RSG pretreatment group (intravenously pre-injected with 100 µg/kg LXA4 for 30 min, and 10 mg/kg RSG for 20 min prior CLP).

### Mortality Rate

The rats (n = 18 per group) were subjected to CLP or sham surgery. The animals were monitored following the surgical procedure and administered the drugs as detailed in the previous section. The time of animal death was recorded. The rats were observed for 24 h after CLP. The mortality rate during this 24 h observation period was calculated.

### Sample Collection

Eighteen hours after CLP, the rats were all anesthetized with isoflurane inhalation. Urine and blood samples were collected from the bladder and abdominal aorta, respectively. The upper section of the right kidney was obtained and fixed in 4% formaldehyde overnight for further examination. Another small segment of the kidney tissue was excised and immediately frozen in liquid nitrogen to prepare frozen sections. Other tissues were collected for ELISA and western blot analyses.

### Detection of Renal Function

The plasma was isolated from blood samples by centrifugation at 2000 × g for 20 min at 4°C and used for measuring blood urea nitrogen (BUN) and serum creatinine (SCr) using a biochemical analyzer (AU640, Watford Olympus, UK). Urinary KIM-1 and NGAL were determined using ELISA kits, according to the manufacturer’s instructions.

### Hematoxylin-Eosin (H&E) Staining

Kidneys were fixed in 4% formaldehyde overnight and then embedded in paraffin. Kidney tissues were cut into 5 μm sections for histopathological analysis using H&E staining. Renal tubular injuries, including tubular epithelial injury, tubule dilation, cast formation, and necrosis, were graded on a scale of 0–4 (0, no change; 1, change affecting < 25% of the field; 2, change affecting 25–50% of the field; 3, change affecting 50–75% of the field; 4, change affecting > 75% of the field).

### Measurement of Inflammatory Markers

The levels of IL-6, TNF-α, HGMB1, and LXA4 in the kidney tissues were determined using ELISA kits, according to the manufacturer’s instructions.

### Cell Culture and LPS Treatment

NRK52E cells (Rat renal proximal tubule cell line) from American Tissue Culture Collection, were cultured in Dulbecco’s Modified Eagle’s medium (DMEM) with 10% FBS. They were kept in a humidified atmosphere with 5% CO2 in air at 37°C. Referred to the method of constructing the *in vitro* septic model, conflucent NRK52E cells were cultured with low glucose, serum-starved DMEM, along with 1 μg/ml lipopolysaccharide (LPS) for 24h with or without 10 nM LXA4 pretreatment for 12 h.

### Senescence-Associated β-Galactosidase

Frozen kidneys that were cut into 5 μm sections, and NRK52E cells planted in sterile cover glasses and then subjected to LPS were probed with a cellular senescence assay kit, according to the manufacturer’s instructions. Notably, in order to avoid detecting endogenous β-galactosidase, the sections were incubated in X-gal staining solution for 1 h as we earlier reported.

### PPAR-γ Transcriptional Activity Assay

The transcriptional activity of PPAR-γ was determined using the PPAR-γ transcription factor assay kit. The cell proteins were extracted and incubated for 15 min at 37°C in wells coated with specific PPAR response element oligonucleotide sequences, then exposed to the primary anti-PPAR-γ polyclonal antibody. Subsequently, the horseradish peroxidase-conjugated secondary antibody and the 3,39,5,59-tetramethylbenzidine substrate were added and the absorbance was quantified at 450 nm using a spectrophotometer.

### Inhibition of PPAR-γ Expression by siRNA Transfection

PPAR-γ-siRNA duplexes targeting PPAR-γ rat gene (GenBank accession ID: 25664) were used to silence PPAR-γ expression. A nonspecific siRNA was taken as siRNA control (NC group). Transfection into NRK52E cells was carried out using Lipofectamine 3000 transfection reagent following the manufacturer**’**s instructions.

### Immunofluorescence Analysis

Kidney sections of 5 μm thickness embedded in paraffin and NRK52E cells planted in sterile cover glasses were stained with the following primary antibodies: PPAR-γ (1:100), p-p65 (1:100), p53 (1:100), p21 (1:100) at 4°C overnight, followed by incubation with anti-rabbit or anti-mouse IgG (1:1000) at 25°C for 2 h. Cell nuclei were stained with 6-diamino-2-phenylindole (DAPI) at 25°C for 5 min. All images were examined using an EVOS FL fluorescence microscope (EVOS FL, Life Technology, US).

### Western Blotting

Western blotting was performed following standard procedures as described using the following primary antibodies: PPAR-γ (1:100), p21 (1:1000) and p53 (1:1000). All western blots were repeated at least three times. Images were acquired using a Tanon 5500 imaging system (Tanon, Shanghai, China). The obtained images were scanned with Image J 1.8.0 (NIH, US), and the data were expressed as values relative to that of the sham group. All the western blot bands are shown in **Supplementary Figure 1**.

### Statistical Analysis

Statistical analysis was performed using SPSS 13.0 (SPSS Inc., Chicago, IL, US) and GraphPad Prism 7.03 (La Jolla, CA, US). The Kolmogorov-Smirnov test was used to test the normality of the data. One-way analysis of variance (ANOVA) was used to make multiple comparisons among different groups. Quantitative data are presented as the mean ± standard error of mean (SE). *P* < 0.05 was considered statistically significant.

## Results

### Changes Found in Septic Renal Injury Were Consistent With Tubular Cell Senescence and Inflammation

**Figure 1:**
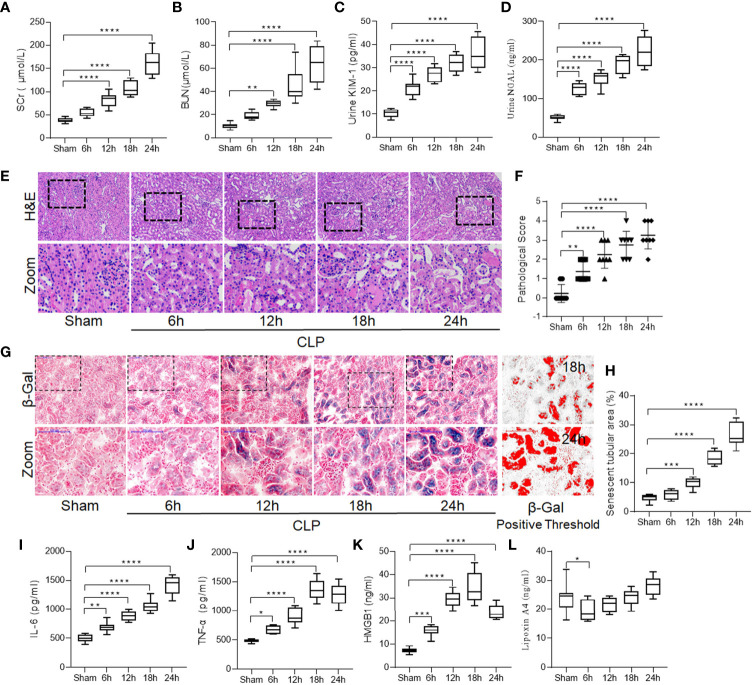
Increased renal damage was accompanied by enhanced tubular cell senescence and inflammation in sepsis induced-AKI. Male Sprague-Dawley rats underwent CLP were sacrificed at the time point of 6-h, 12-h, 18-h, 24-h after surgery. Renal function alternation, renal necrosis and renal tubular epithelial cells senescence were detected with different methods in kidneys. **(A–D)** levels of SCr, BUN, urinary KIM-1 and NGAL at different surgery time points after CLP. **(E)** Renal damage of rats at different surgery time points after CLP (H&E; scale bar 100 μm). **(F)** Kidney histopathology evaluation scores at different surgery time points. **(G)** Representative images of cellular senescence in kidneys after CLP (β-Gal staining; scale bar 100 μm); Red: β-Gal positive threshold. **(H)** Senescent tubular areas at different surgery time points after CLP. **(I–K)** levels of IL-6, TNF-α and HMGB1 at different surgery time points after CLP. **(L)** levels of lipoxin A4 at different surgery time points after CLP. Data are presented as mean ± SE (n = 8). *p < 0.05; **p < 0.005; ***p < 0.001; ****p < 0.0001; CLP, cecal ligation and puncture; AKI, acute kidney injury; H&E, hematoxylin–eosin staining; SCr, serum creatinine; BUN, blood urea nitrogen; KIM-1, kidney injury molecule-1; NGAL, neutrophil gelatinase-associated lipocalin; IL-6, interlecukin-6; IL-8, interlecukin-8; TNF-α, tumor necrosis factor-alpha; LXA4, lipoxin A4.

Rats with similar weight (**Supplementary Table S1**) that underwent CLP exhibited significant renal dysfunction over time, as reflected by a gradual increase in the levels of SCr, BUN, urinary KIM-1, and NGAL (all *P* < 0.05; **Figures 1A–D**). Renal pathological damage was also detected consistent with the functional injury. Tubular necrosis, tubular epithelial swelling, and vacuolar degeneration were observed over time, especially at 18 and 24 h post CLP (**Figures 1E, F**).

Consistent with septic renal injury, we observed that renal tubular epithelial cell senescence increased significantly in the CLP group compared to the sham group, and the percentage of β-gal-positive area increased over time (**Figures 1G, H**), which prompted us to consider senescence as an important cause of renal injury induced by sepsis.

Since an imbalance between the pro- and anti-inflammatory systems is considered to be a vital mechanism of sepsis (21), we measured the levels of pro-inflammatory cytokines including IL-6, TNF-α, and HMGB1 in the kidneys. The results showed that CLP significantly increased the levels of IL-6, TNF-α, and HMGB1 over time, and this mirrored the pattern of renal injury and cellular senescence (all *P* < 0.05; **Figures 1I–K**). However, the level of LXA4, the main mediator of inflammatory resolution, did not significantly increase over time (**Figure 1L**), indicating that CLP decreased the resolution of inflammation.

### Inhibition of Senescence Restored Renal Function and Attenuated Septic Inflammatory Response

**Figure 2:**
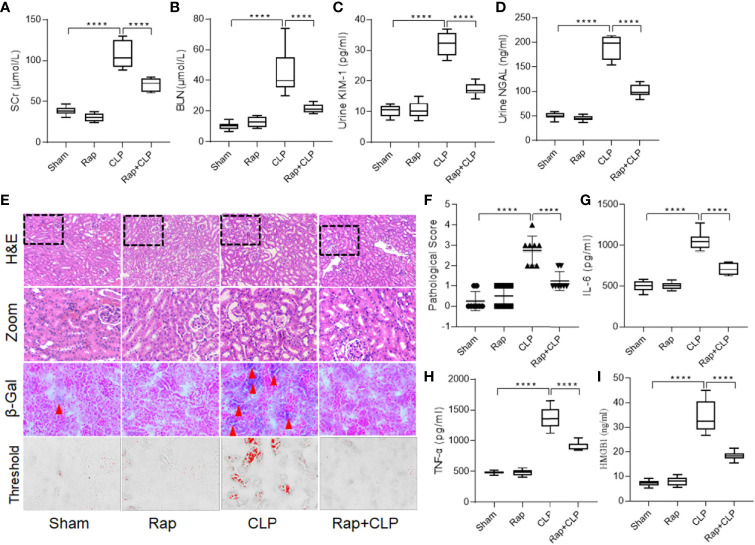
Rapamycin restores renal function and attenuates septic inflammatory response. Male Sprague-Dawley rats were treated with or without rapamycin 1 mg/kg *i.p.* for 1h before CLP and were sacrificed at the time point of 18-h after surgery. Renal function alternation, renal necrosis and inflammatory cytokines were detected. **(A–D)** levels of SCr, BUN, urinary KIM-1 and NGAL among the four groups. **(E)** Renal damage and senescence of rats among the four groups (H&E; scale bar 100 μm). **(F)** Kidney histopathology evaluation scores among the four groups. **(G–I)** levels of IL-6, TNF-α and HMGB1 in kidneys among the four groups. Data are presented as mean ± SE (n = 8). ****p < 0.0001; CLP, cecal ligation and puncture; H&E, hematoxylin–eosin staining; SCr, serum creatinine; BUN, blood urea nitrogen; KIM-1, kidney injury molecule-1; NGAL, neutrophil gelatinase-associated lipocalin; IL-6, interlecukin-6; IL-8, interlecukin-8; TNF-α, tumor necrosis factor-alpha.

To explore the role of cellular senescence in septic renal injury, a senescence inhibitor, rapamycin (1 mg/kg, *i.p.*) was used to pretreat the rat before constructing the sepsis model. As shown in **Figures 2A–D**, administration of rapamycin significantly restored septic renal function, as detected by the decreased SCr, BUN, urinary KIM-1, and NGAL levels at 18 h after construction of the CLP model (**Figures 2A–D**, all *p* < 0.001, CLP *vs.* Rap + CLP group). **Figure 2E** shows representative micrographs of each group. Administration of rapamycin significantly inhibited renal tubular cell senescence. In addition, compared to the sham group, the kidneys in the CLP group showed significant histopathological changes, characterized by tubule dilation, vacuolar degeneration, and necrotic tubules. Administration with rapamycin minored the pathological changes, compared to that in the CLP group (**Figure 2F**, *p* < 0.001, CLP *vs.* Rap + CLP group). Furthermore, pretreatment with rapamycin also attenuated the septic inflammatory response, as reflected by a significant decrease in IL-6, TNF-α, and HMGB1 levels (**Figures 2G–I**, all *p* < 0.001, CLP *vs.* Rap + CLP group). These results indicate that cellular senescence plays an important role in the progression of septic inflammatory response and kidney injury.

### Exogenous LXA4 Supplementation Alleviated Septic AKI

**Figure 3:**
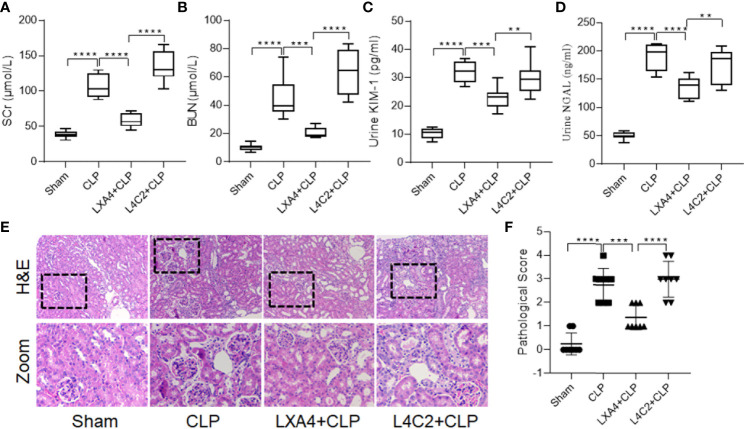
LXA4 alleviates septic AKI. Male Sprague-Dawley rats were treated with or without LXA4 (100 µg/kg, *i.p.*) for 30-min or BOC-2 (50 mg/kg, *i.p.*) for 20min before CLP and were sacrificed at the time point of 18-h after surgery. Renal function alternation and renal necrosis were detected. **(A–D)** levels of SCr, BUN, urinary KIM-1 and NGAL among the four groups. **(E)** Renal damage of rats among the four groups (H&E; scale bar 100 μm). **(F)** Kidney histopathology evaluation scores among the four groups. Data are presented as mean ± SE (n = 8). **p < 0.01; ***p < 0.001; ****p < 0.0001; LXA4, Lipoxin A4; CLP, cecal ligation and puncture; H&E, hematoxylin–eosin staining; SCr, serum creatinine; BUN, blood urea nitrogen; KIM-1, kidney injury molecule-1; NGAL, neutrophil gelatinase-associated lipocalin.

To further explore the renoprotective effect of LXA4 on AKI induced by sepsis, rats were pretreated with LXA4 (100 µg/kg, *i.v.*) prior to CLP surgery. As shown in **Supplementary Figure 2**, LXA4 preconditioning significantly increased its level in renal tissue, as determined by ELISA. Furthermore, exogenous LXA4 supplementation reduced renal dysfunction induced by sepsis, as evidenced by lower levels of serum BUN, SCr, urinary KIM-1, NGAL, and pathological scores than that in the CLP groups (**Figure 3**; all *p* < 0.001, CLP *vs.* LXA4 + CLP group). Furthermore, the LXA4 antagonist, Boc-2, partly reversed the effects of LXA4, as evidenced by worsening of the pathological abnormalities and increased pathological score, BUN, SCr, urinary KIM-1, and NGAL levels (**Figure 3**; all *p* < 0.001, LXA4 + CLP *vs.* L4C2 + CLP group). These results indicate that LXA4 preconditioning confers protective effects against septic AKI.

### LXA4 Preconditioning Alleviated Renal Inflammation and Tubular Epithelial Cell Senescence

**Figure 4:**
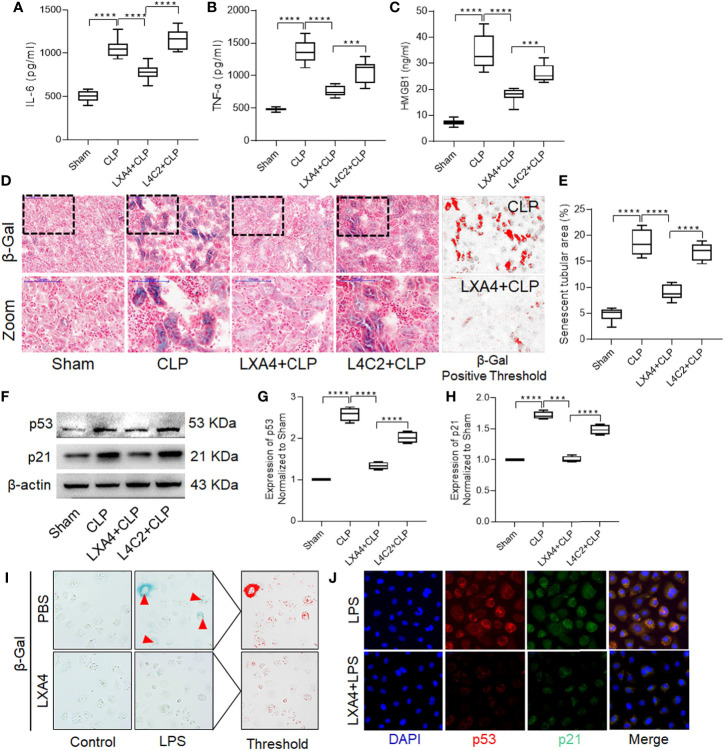
LXA4 alleviates renal inflammation and tubular epithelial cell senescence. Levels of inflammatory cytokines and cellular senescence were detected. **(A–C)** levels of IL-6, TNF-α and HMGB1 at among the four groups. **(D)** Representative images of cellular senescence in kidneys after CLP (β-Gal staining; scale bar 100 μm); Red: β-Gal positive threshold. **(E)** quantitative analysis of senescent tubular area among the four groups. **(F–H)** expression of p53 and p21 protein in kidney tissues among the four groups. **(I)** Representative images of cellular senescence in NRK52E cells after LPS treatment (β-Gal staining). **(J)** Expression of p53 and p21 protein in NRK52E cells after LPS treatment. Data are presented as mean ± SE (n = 8). ***p < 0.001; ****p < 0.0001; CLP, cecal ligation and puncture; IL-6, interlecukin-6; IL-8, interlecukin-8; TNF-α, tumor necrosis factor-alpha; LPS, lipopolysaccharide.

To explore the underlying mechanism of LXA4, we first determined the levels of cytokines in kidney tissues. Pretreatment with LXA4 significantly attenuated the septic inflammatory response, as reflected by the significant decrease in IL-6, TNF-α, and HMGB1 levels (**Figures 4A–C**, all *p* < 0.001, CLP *vs.* LXA4 + CLP group). Furthermore, we also analyzed β-gal staining and expression of the p53/21 senescence pathway to determine the effect of LXA4 on cellular senescence. As shown in **Figure 4D**, the kidney sections from the CLP model expressed significant more β-gal positive areas, and LXA4 administration significantly inhibited renal tubular epithelial cell senescence (**Figure 4E**, *p* < 0.001, CLP *vs.* LXA4 + CLP group). The changes in the expression of the senescence biomarkers, p53, and p21, were also consistent with the pattern of β-gal staining in the kidney tissues (both *p* < 0.001, CLP *vs.* LXA4 + CLP group, **Figures 4F–H**). Moreover, we found LPS administration led to significant senescence in NRK52E cells and LXA4 administration significantly inhibited NRK52E cell senescence (**Figure 4I**) and the expression of the senescence biomarkers, p53 and p21 (**Figure 4J**).

### LXA4 Preconditioning Attenuated Septic AKI and Increased the Survival Rate of the Rats in a PPAR-γ-Dependent Manner

**Figure 5:**
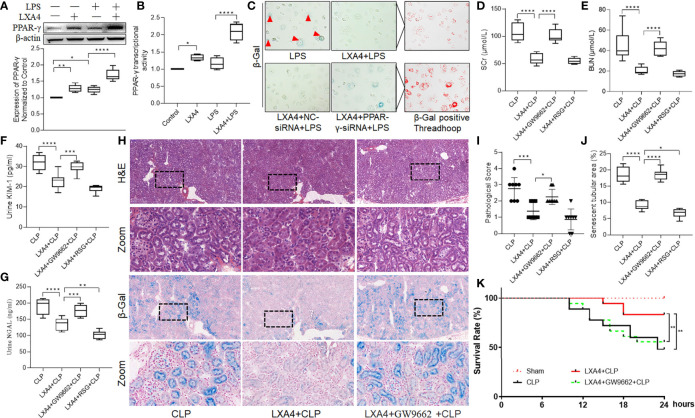
LXA4 attenuates AKI and increased rat survival rate in a PPAR-γ-dependent manner. NRK52E cells were treated with 1 μg/ml LPS for 24h with or without 10 nM LXA4 pretreatment for 12h, and PPAR-γ-siRNA transfection for 48h. Male Sprague-Dawley rats were treated with or without LXA4 (100 µg/kg, *i.p.*) for 30-min, or GW9662 (1 mg/kg, *i.v.*) or RSG (10 mg/kg, *i.v.*) for 20min prior CLP and were sacrificed at the time point of 18-h after surgery. Renal function alternation, renal pathological injury and senescence in the neighbored section were detected. **(A)** expression of PPAR-γ among the four groups. **(B)** PPAR-γ transcriptional activity among the four groups. **(C)** Representative images of NRK52E cell senescence among the four groups. **(D–G)** levels of SCr, BUN, urinary KIM-1 and NGAL among the four groups. **(H)** Representative images of renal damage and cellular senescence in the neighbored kidney section among the four groups (H&E and β-Gal staining; scale bar 100 μm). **(I, J)** Kidney histopathology scores and senescent tubular area among the four groups. **(K)** Survival rate after LXA4 treatment. The survival rate was observed for 24-h after the CLP operation, n=18. **(A–G)** Data are presented as mean ± SE (n = 8). *p < 0.05; **p < 0.005; ***p < 0.001; ****p < 0.0001; LXA4, Lipoxin A4; RSG, Rosiglitazone; CLP, cecal ligation and puncture; H&E, hematoxylin–eosin staining; SCr, serum creatinine; BUN, blood urea nitrogen; KIM-1, kidney injury molecule-1; NGAL, neutrophil gelatinase-associated lipocalin.

As PPAR is one of the main receptors of LXA4, and LXA4 was reported to mediate PPAR-γ-dependent resolution of inflammation (20), we then tested the expression of and activity of PPAR-γ in NRK52E cells. As shown in **Figures 5A, B**, LXA4 administration significantly enhanced PPAR-γ expression and upregulated PPAR-γ activity. Moreover, we found that the anti-senescent effect of LXA4 was abolished when we silenced PPAR-γ expression with PPAR-γ-siRNA (**Figure 5C**), indicating that LXA4 preconditioning attenuated cell senescence in a PPAR-γ-dependent manner.

To further confirmed the results, we pretreated the rats with a specific PPAR-γ antagonist, GW9662, before LXA4 administration. As shown in **Figures 5D–I**, the protective effect of LXA4 in reducing septic renal dysfunction was inhibited by GW9662, as evidenced by higher serum BUN and SCr, urinary KIM-1, and NGAL levels, and pathological scores in LXA4 + GW9662 + CLP group compared to those in LXA4 + CLP group (all *p* < 0.001). Furthermore, we analyzed tubular senescence in the same areas of the kidney sections as the pathological visual field. As shown in **Figure 5H**, we found that the tubular cell senescence was consistent with the pathological injury, and the anti-senescence effect of LXA4 was abolished by GW9662 (**Figure 5J**, *p* < 0.001, LXA4 + CLP *vs.* LXA4 + GW9662 + CLP). Furthermore, PPAR-γ activator RSG enhanced the protective effect of LXA4, as reflected by lower urinary NGAL level and percentage of senescent tubular areas (**Figures 5G, J**, both *p* < 0.05, LXA4 + CLP *vs.* LXA4 + RSG + CLP). We also assayed the survival rate of the rats in the study, and found that sepsis AKI led to decrease in the survival rate at 18 h after CLP (44%, *p* < 0.05, CLP *vs.* Sham group, **Figure 5K**). LXA4 significantly increased the survival rate of the rats (83%, *p* < 0.05, CLP *vs.* LXA4 + CLP group), and its effect was reversed by GW9662 (72%, *p* < 0.05, LXA4 + GW9662 + CLP *vs.* LXA4 + CLP group). These results indicate that LXA4 preconditioning attenuates septic renal inflammation and cell senescence in a PPAR-γ-dependent manner.

### LXA4 Attenuated Septic AKI *via* Inhibition of PPAR-γ/NF-κB Pathway

**Figure 6:**
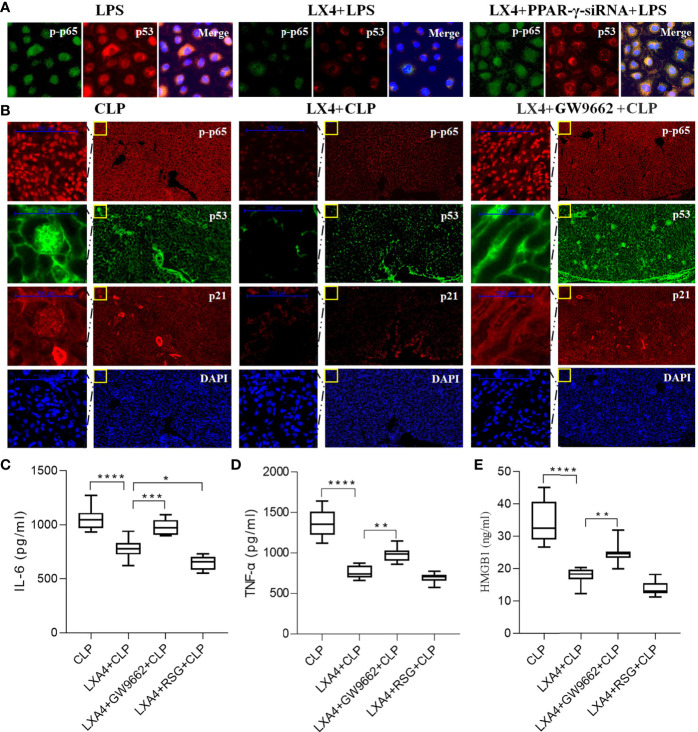
LXA4 attenuates septic AKI *via* inhibition of PPAR-γ/NF-κB pathway. Expression of p-p65, p53 and p21 in the neighbored section and levels of inflammatory cytokines in NRK52E cells and kidneys were also detected. **(A)** Expression of p-p65 and p53 in the NRK52E cells were detected by IF. **(B)** Expression of p-p65, p53 and p21 in the neighbored kidney sections were detected by IF. **(C–E)** levels of IL-6, TNF-α and HMGB1 at among the four groups. Data are presented as mean ± SE (n = 8). *p < 0.05; **p < 0.005; ***p < 0.001; ****p < 0.0001; LXA4, Lipoxin A4; RSG, Rosiglitazone; CLP, cecal ligation and puncture; IL-6, interlecukin-6; IL-8, interlecukin-8; TNF-α, tumor necrosis factor-alpha.

NF-κB has been found to regulate both the inflammatory pathway and p53/p21 senescence pathway (22), and LXA4 reduces NF-κB-mediated transcriptional activation (23). To explore whether LXA4 reduces NF-κB-mediated transcriptional activation in a PPAR-γ-dependent manner, we then silenced PPAR-γ expression with PPAR-γ-siRNA and tested the activation of NF-κB p65 (p-p65) and p53 expression in NRK52E cells. We found that PPAR-γ knockdown significantly reversed the effect of LXA4 (**Figure 6A**).

To further confirm the results, we analyzed the expression of p65/p53/p21 pathway in the same kidney sections. As shown in **Figure 6B**, LXA4 pretreatment significantly inhibited the activation of NF-κB p65 (p-p65), p53, and p21, and the changes were reversed by GW9662. Moreover, the levels of inflammatory cytokines including IL-6, TNF-α, and HMGB in LXA4 + GW9662 + CLP group were all higher compared to that in LXA4 + CLP group (all *p* < 0.05, **Figures 6C–E**), indicating that the anti-inflammatory effect of LXA4 was abolished by the PPAR-γ antagonist, GW9662. In contrast, RSG enhanced the anti-inflammatory effect of LXA4, as reflected by lower IL-6 level (**Figure 6C**, *p* < 0.05, LXA4 + CLP *vs.* LXA4 + RSG + CLP). These results indicate that LXA4 inhibits the NF-κB pathway in a PPAR-γ-dependent manner.

## Discussion

**Figure 7:**
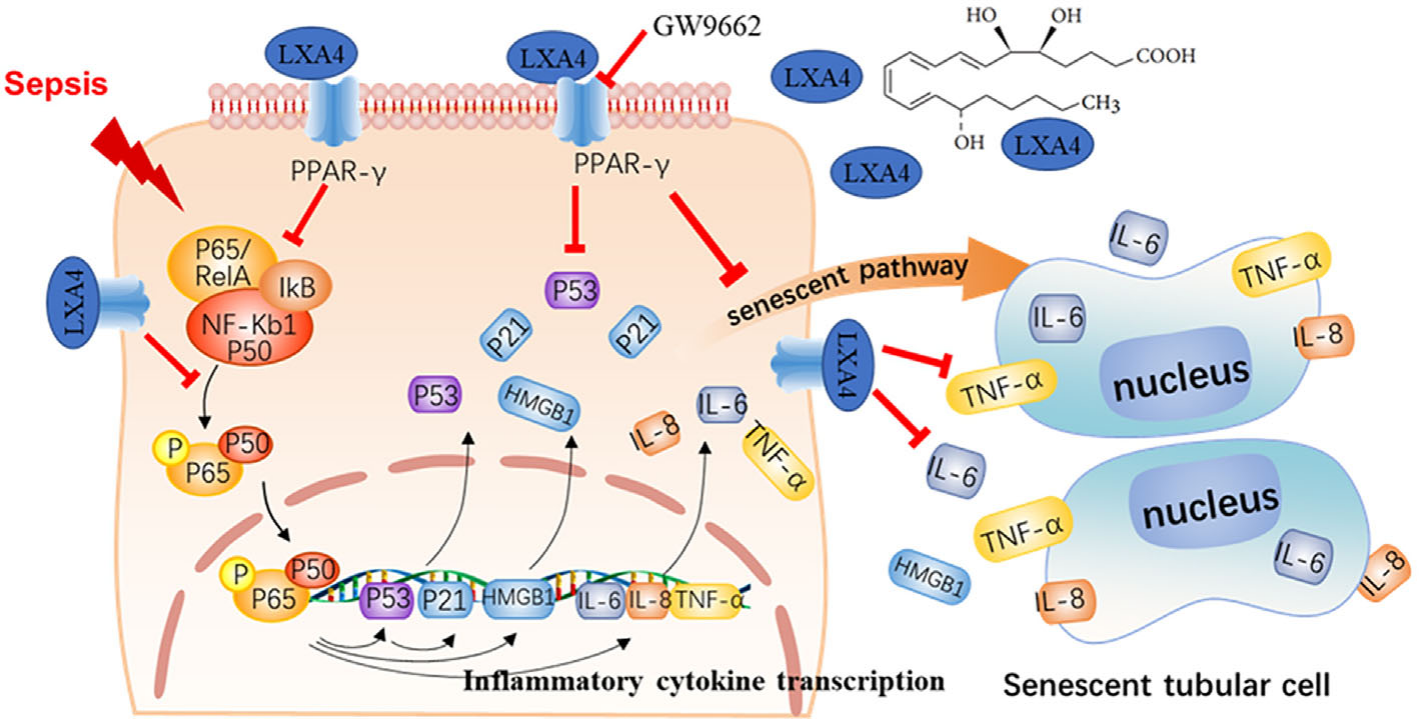
LXA4 attenuates septic AKI *via* blocking crosstalk between inflammation and premature senescence in PPAR-γ-dependent manner. Sepsis would cause activation of NF-κB-mediated inflammation response and NF-κB/p53 mediated cellular premature senescence in kidney tissues. The inflammation response interplays with cellular premature senescence in septic AKI. Pretreatment with LXA4 promotes inflammation resolution and blocks the crosstalk between inflammation and premature senescence *via* PPAR-γ/NF-κB signaling.

Sepsis-induced multiple organs failure is characterized by an imbalance between the pro- and anti-inflammatory systems (21). AKI occurs in more than 50% of patients with septic shock, and its associated mortality is unacceptably high (24). Unfortunately, effective preventive treatments are still lacking (20). In this investigation, we established a CLP model to mimic sepsis *in vivo*, and AKI was found to occur in the early stage of sepsis. Our results show that the crosstalk between the excessive inflammatory response and tubular epithelial cell senescence takes part in septic AKI. Pretreatment with LXA4 significantly restored renal function, increased rat survival rate, and inhibited NF-κB-mediated inflammatory response and premature senescence. Furthermore, the protective effect of LXA4 was reversed by the PPAR-γ siRNA and antagonist *via* regulation of the downstream NF-κB network (**Figure 7**). These results indicated that LXA4 exerted its renoprotective effects by blocking the crosstalk between inflammation and premature senescence in a PPAR-γ-dependent manner.

Cellular senescence, a stable cell cycle arrest, is implicated in both body aging and many acute pathological processes. Major triggers of cellular senescence include telomere shortening (also referred to as replicative senescence), and stressors such as oxidative stress and inflammation (referred to as SIPS) (25). Although our previous study reported that sepsis causes cellular senescence in the kidneys (8), its underlying mechanism has not been explored very well. Therefore, in this investigation, we further examined the role of senescence in septic AKI and explored in depth the possible mechanisms and therapeutic implications.

In this investigation, inflammation and tubular epithelial cell senescence were found to progress consistently over time following the CLP surgery, and their dynamic changes mirrored the patterns of pathological and functional injury of the kidney. We then inhibited senescence with rapamycin and found that the treatment improved renal function and alleviated the inflammatory response. Furthermore, promoting the resolution of inflammation with LXA4 simultaneously alleviated cellular senescence, indicating that inflammation plays an important role in the pathogenesis of septic AKI. Accumulating evidence suggests a potential crosstalk between inflammation and senescence in septic AKI. First, systemic inflammation in sepsis can lead to premature senescence in different tissues, including the lung (10), coronary arteries (11), and brain (12). Increased inflammation and sub-optimal resolution also act as drivers for several aging-associated kidney pathologies and senescence (26, 27). Conversely, kidney senescence was found to be associated with increased severity of septic AKI and increased inflammatory response (14, 15). Senescent cells are thought to accumulate in the kidneys and compromise renal function, essentially caused by the unique property of senescent cells to secrete a number of pro-inflammatory and damage-inducing molecules, commonly referred to as the SASP. To the best of our knowledge, this is the first study to report a crosstalk between inflammation and senescence in septic AKI, and to propose a novel mechanism for potential therapeutic targeting.

LXA4 is a member of the lipoxin family that was identified as the first endogenous “braking signals” or “stop signals” of inflammation (28). LXA4 was also reported to augment host defense (29), reduce blood bacterial load, increase macrophage recruitment, and reduce systemic inflammation in sepsis (30). Sheng-Hua Wu showed that LXA4 may promote resolution of acute inflammation in renal IR injury *via* activation of the p38 MAPK/PPAR-γ/Nrf2-ARE/HO-1 pathway (20). Recent studies have indicated that LXA4 plays a potential role in the prevention of aging and inflammation-associated senescence (26, 31). Our study showed that the level of LXA4 did not significantly increase after CLP, and this contributed to defective inflammation resolution. Conversely, LXA4 treatment could regulate inflammation mitigation and block the crosstalk between inflammation and senescence.

PPAR-γ is an isoform of PPAR that belongs to a super family of nuclear receptors. PPAR-γ receptor is found to play a crucial role in the modulation of senescence-associated fatty acid homeostasis and inflammation (32). To explore whether LXA4 mediates inflammation resolution in a PPAR-γ-dependent manner in septic AKI, we pretreated the rat with PPAR-γ antagonist GW9662. Our results showed that the protective effect of LXA4 was reversed by GW9662, indicating that LXA4 exerts its renal protection by binding to PPAR-γ. As the main function of PPAR during the inflammatory reaction is to promote the inactivation of NF-κB (23), and NF-κB signaling was also implicated in cellular senescence *via* the ROS‐NF‐κB‐p53 pathway (33), we further determined the expression of the NF‐κB‐p53-p21 senescent pathway in the kidney sections and found that the pathway was activated in the CLP model, and the changes were reversed by the PPAR-γ antagonist GW9662. These results are consistent with a recent study showing that PPAR-γ agonists delays cellular senescence and age-associated metabolic disease (34). All the results indicate that LXA4 exerts its anti-senescence and anti-inflammatory effects *via* the PPAR-γ/NF-κB pathway.

### Limitations of the Study

This study has some limitations that need to be considered. First, consistent with earlier reports, we found that there is no significant senescence in the glomerulus area, and therefore we only focused on renal tubular changes. However, it has been reported that sepsis may cause both tubular and glomerular injuries. Hence, further studies are required to confirm our results, and the effect of LXA4 on glomerular changes should also be explored in the future. Second, the effect of LXA4 on the PPAR-γ/NF-κB signaling pathway was clarified in this study; however, further studies are needed to determine the effect of LXA4 on other anti-inflammatory or anti-senescence pathways, including the TLR4/MyD88 and p16(INK4a) pathways. Third, we did not explore the interaction between renal tubular cell senescence and inflammatory cell activation *in vitro*, and further studies are needed to confirm the crosstalk between inflammation and premature senescence.

### Conclusions

This study conducted a series of *in vivo* and *in vitro* studies to demonstrate that crosstalk between the inflammatory response and premature senescence plays a critical role in septic AKI. LXA4 restored septic renal function and increased the survival rate by inhibiting the NF-κB network in a PPAR-γ-dependent manner.

## Data Availability Statement

The original contributions presented in the study are included in the article/**Supplementary Material** further inquiries can be directed to the corresponding authors.

## Ethics Statement

The animal study was reviewed and approved by Institutional Animal Care and Use Committee at Third Affiliated Hospital of Sun Yat-Sen University.

## Author Contributions

CC and RQ contributed equally to this study. Conceived and designed the experiments: CC, ZH, and HJ. Performed the experiments: CC, RQ, JY, QZ, GS, and XG. Analyzed the data: RQ and XG. Contributed reagents/materials/analysis tools: CC and ZH. Wrote the paper: CC and ZH. All authors contributed to the article and approved the submitted version.

## Funding

This work was, in part, supported by the Postdoctoral Science Foundation of China (Grant No. 2019M663260), the Fundamental Research Funds for the Central Universities of China (Grant No. 20ykpy20), Basic and Applied Basic Research Foundation of Guangdong Province (Grant No. 2019A1515110020), and Medical Research Foundation of Guangdong Province (Grant No. A2020549).

## Conflict of Interest

The authors declare that the research was conducted in the absence of any commercial or financial relationships that could be construed as a potential conflict of interest.
