# Supplementary material for: Lipoxin A4 Restores Septic Renal Function via Blocking Crosstalk Between Inflammation and Premature Senescence
Source: Front Immunol. 2021 Apr 15;12:637753. doi: 10.3389/fimmu.2021.637753 (PMC8084287; doi:10.3389/fimmu.2021.637753)
Supplement: Supplementary file 1 [file DataSheet_1.docx]

Supplementary Material

**Supplementary Table 1:** **Rats body weights among different groups.**

Note: Data are represented as the mean ± standard deviation, n = 8 for each group. Male SD Rats were sacriﬁced at the time point of 6-h, 12-h, 18-h, 24-h after establishment of CLP model.

| **Group** | **Weight (g)** |
| --- | --- |
| Sham | 234.25±9.09 |
| 6h | 235.37±10.22 |
| 12h | 238.62±6.50 |
| 18h | 230.50±9.27 |
| 24h | 236.75±8.41 |

**Supplementary Figure 1.**


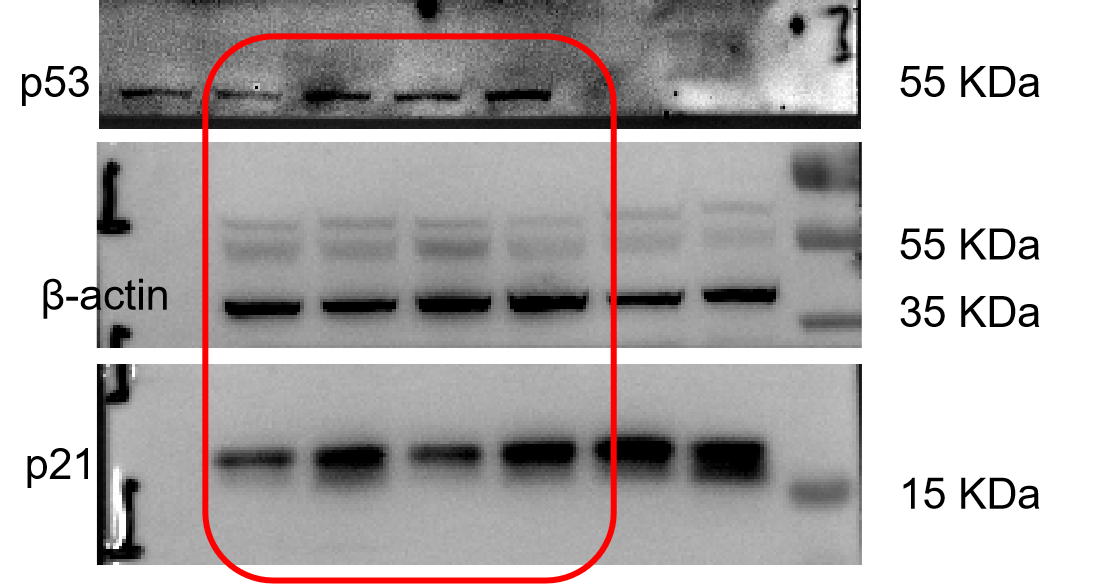


**Supplementary Figure 1.** **Entire blot lanes including molecular weight markers for all cropped Western blot bands shown in the main body of the manuscript.**

The lanes in the rectangle are the exact ones in the main figure.

**Supplementary Figure 2.**

**

**

**Supplementary Figure 2.** **Exogenous LXA4 supplementation increased its level in renal tissue.**

Expression of LXA4 protein in kidney tissues with or without LXA4 pretreatment, detected by ELISA. Male Sprague-Dawley rats were treated with or without LXA4 (100 𝜇g/kg, *i.p.*) for 30-min or BOC-2 (50 mg/kg, *i.p.*) for 20min before CLP and were sacriﬁced at the time point of 18-h after surgery. Data are presented as mean ± SE (n = 8). *****p* < 0.0001; LXA4, Lipoxin A4; CLP, cecal ligation and puncture;
